# Metagenomic analysis reveals differences in the co-occurrence and abundance of viral species in SARS-CoV-2 patients with different severity of disease

**DOI:** 10.1186/s12879-022-07783-8

**Published:** 2022-10-19

**Authors:** Pavel Iša, Blanca Taboada, Rodrigo García-López, Celia Boukadida, José Ernesto Ramírez-González, Joel Armando Vázquez-Pérez, Alejandra Hernández-Terán, José Ángel Romero-Espinoza, José Esteban Muñoz-Medina, Concepción Grajales-Muñiz, Alma Rincón-Rubio, Margarita Matías-Florentino, Alejandro Sanchez-Flores, Edgar Mendieta-Condado, Gisela Barrera-Badillo, Susana López, Lucía Hernández-Rivas, Irma López-Martínez, Santiago Ávila-Ríos, Carlos F. Arias

**Affiliations:** 1 Departamento de Genética del Desarrollo y Fisiología Molecular, Instituto de Biotecnología, Universidad Nacional Autónoma de México, Cuernavaca, Morelos Mexico; 2 Centro de Investigación en Enfermedades Infecciosas, Instituto Nacional de Enfermedades Respiratorias Ismael Cosío Villegas, Mexico City, Mexico; 3 Instituto de Diagnóstico y Referencia Epidemiológicos, Dirección General de Epidemiología, Ciudad de Mexico, Mexico; 4 Instituto Nacional de Enfermedades Respiratorias Ismael Cosío Villegas, Mexico City, Mexico; 5 Coordinación de Calidad de Insumos y Laboratorios Especializados, Instituto Mexicano del Seguro Social, Mexico City, Mexico; 6 Unidad Universitaria de Secuenciación Masiva y Bioinformática, Instituto de Biotecnología, Universidad Nacional Autónoma de México, Cuernavaca, Morelos Mexico

**Keywords:** SARS-CoV-2, Respiratory viruses, Metagenomic sequencing, COVID-19, Disease severity

## Abstract

**Background:**

SARS-CoV-2 infections have a wide spectrum of clinical manifestations whose causes are not completely understood. Some human conditions predispose to severe outcome, like old age or the presence of comorbidities, but many other facets, including coinfections with other viruses, remain poorly characterized.

**Methods:**

In this study, the eukaryotic fraction of the respiratory virome of 120 COVID-19 patients was characterized through whole metagenomic sequencing.

**Results:**

Genetic material from respiratory viruses was detected in 25% of all samples, whereas human viruses other than SARS-CoV-2 were found in 80% of them. Samples from hospitalized and deceased patients presented a higher prevalence of different viruses when compared to ambulatory individuals. Small circular DNA viruses from the *Anneloviridae* (Torque teno midi virus 8, TTV-like mini virus 19 and 26) and Cycloviridae families (Human associated cyclovirus 10), Human betaherpesvirus 6, were found to be significantly more abundant in samples from deceased and hospitalized patients compared to samples from ambulatory individuals. Similarly, *Rotavirus A*, *Measles morbillivirus* and *Alphapapilomavirus 10* were significantly more prevalent in deceased patients compared to hospitalized and ambulatory individuals.

**Conclusions:**

Results show the suitability of using metagenomics to characterize a broader peripheric virological landscape of the eukaryotic virome in SARS-CoV-2 infected patients with distinct disease outcomes. Identified prevalent viruses in hospitalized and deceased patients may prove important for the targeted exploration of coinfections that may impact prognosis.

**Supplementary Information:**

The online version contains supplementary material available at 10.1186/s12879-022-07783-8.

## Introduction

The World Health Organization declared SARS-CoV-2 a world pandemic on March 11th, 2020. Since it was first reported in late 2019 in Wuhan, China, it has spread worldwide. SARS-CoV-2 is the etiologic agent of the respiratory disease COVID-19, which can have different pathological presentations, from asymptomatic and mild respiratory illness to severe pneumonia with high fatality rates. Different factors, including age [1], presence of comorbidities [2–4], viral genetic factors [5, 6], host genetic constellations [7], and even coinfection with other pathogens and the composition of the microbiome [8], have been reported to affect the outcome and severity of the disease. In Mexico, there is a high prevalence of several comorbidities associated with the severity of COVID-19, specifically overweight and obesity, which are associated with diabetes type 2 and high blood pressure [2, 4, 9].

Since the first observation of the new coronavirus, there have been reports of the occasional presence of coinfections with other viruses [10, 11], and some of these coinfections have been reported in patients with a severe pathological course [12, 13]. However, despite the importance of the presence of other viruses, data on viral coinfections remain limited, focusing primarily on specific respiratory viruses through RT-PCR assays [10–20], and few studies have characterized the samples using high-throughput sequencing [21–25]. Some studies have reported a relatively high presence of respiratory viruses other than SARS-CoV-2, with 20–45% of samples being positive for other respiratory viruses [12, 19, 22]; however, generally, low levels of coinfections have been reported (less than 5% of samples containing other respiratory viruses) [10, 14, 17, 20, 26, 27].

We characterized respiratory samples from 120 subjects with different clinical symptoms in the present study using a classical metagenomic random-amplification (shotgun) method. The global analysis of viral populations showed many different human viral species and viruses from other origin such as plants and insects. We observed differences in the abundance of human viral species when samples from ambulatory, hospitalized, and deceased individuals were compared, being higher in hospitalized and deceased individuals compared to ambulatory patients.

## Methods

### The ethical statement, sample collection, and diagnostics

Samples and metadata collected for this work are considered part of the national response to COVID-19 and are directly related to prevention and disease control. Samples used were collected between the 13th of March and the 1st of May 2020 and processed under the Mexican Official NOM-017-SSA2-2012 (http://sersalud.cdmx.gob.mx/portalut/archivo/Art121FI/Normatividad_SSPDF/NOM-017-SSA2-2012.pdf) for epidemiological surveillance of Viral Respiratory Disease, emitted and approved by the CONAVE (National Counsel of Epidemiology Surveillance) of the Ministry of Health of the Government of Mexico, and based on this norm ethical approval is not required. Clinical samples were collected at the “Instituto de Diagnóstico y Referencia Epidemiológicos” (InDRE), and “Instituto Nacional de Enfermedades Respiratorias Ismael Cosio Villegas” (INER), by guidance and regulations of declaration of Helsinki, as part of the early diagnostics scheme in public health laboratories and hospitals in Mexico City (Red Nacional de Laboratorios Estatales de Salud Pública, RNLSP; Instituto Nacional de Enfermedades Respiratorias, INER; and Instituto Mexicano del Seguro Social, IMSS). Based on the Mexican Official NOM-017-SSA2-2012 informed consent from patients was not required. All samples were anonymized before use.

Oro- and/or nasopharyngeal swabs, as well as tracheal aspirates were collected and placed in virus transport medium upon collection. The diagnosis was done using validated protocols for SARS-CoV-2 RNA detection, as approved by InDRE and by the World Health Organization (WHO). Depending on the indications of medical staff, in some cases, a panel of respiratory viruses xTAG RVPv1 (Luminex Molecular Diagnostics, Austin, TX) or only influenza virus detection: H1N1pdm09 (https://www.who.int/csr/resources/publications/swineflu/CDCRealtimeRTPCR_SwineH1Assay-2009_20090430.pdf), H3N2 and Influenza B (https://www.who.int/influenza/gisrs_laboratory/CDC_Laboratory_Support_for_Influenza_Surveillance_Info_Sheet_Aug2017.pdf) were used for additional screening and virus identification. We have included samples obtained from three different types of individuals: deceased, hospitalized (severe condition), or ambulatory patients. The demographic and clinical characteristics of the participants, such as age, sex, and comorbidities, are included in the Additional file 1: Table S1.

### Sample processing and whole metagenome sequencing

Clinical samples were extracted in BSL2 or BSL3 laboratories using required biosafety operational standards, including respirators, protective clothing, and head and eye protection. All samples were prepared for RNA extraction as described previously [28]. Briefly, centrifuged and 0.45 μm filtered supernatants were treated with Turbo DNase and RNAse. Nucleic acids were extracted using the PureLink™ Viral RNA/DNA Kit (ThermoFisher), or QIAamp viral RNA minikit (Qiagen). Total cDNA was synthesized using the SuperScript III Reverse Transcriptase System (ThermoFisher) and primer A1 (5′-GTTTCCCAGTAGGTCTCN9-3′) or primer B1 (5′-GCCGGAGCTCTGCAGATATCN9-3′), both of which contained a degenerated 9-mer sequence at the 3′ end. The second strand was generated by two rounds of synthesis with Sequenase 2.0 (Affymetrix, USB, Ohio, USA), or Klenow fragment polymerase (New England Biolabs), followed by 15 cycles of amplification using Phusion DNA polymerase with primer A2 (5′-GTTTCCCAGTAGGTCTC-3′), or 25 cycles of amplification using Expand High Fidelity DNA polymerase (Roche) and primer B2 (5′-GCCGGAGCTCTGCAGATATC-3′). Primers A2 and B2 contain specific sequences of primers A1 and B1, which were used to prepare cDNA, and therefore amplify only particular products produced by the A1 ad B1 primers. Purified dsDNA was used as input to generate whole-metagenome sequencing libraries using Nextera XT DNA library preparation kits (Illumina) (https://support.illumina.com/sequencing/sequencing_kits/nextera_xt_dna_kit/documentation.html). Finally, the samples were sequenced on an Illumina NextSeq 500 platform using 2 × 75-cycle or 2 × 150-cycle high-output kits to obtain paired-end reads. Sequencing yields are reported in the Additional file 1: Table S2.

### Metagenomic data analysis

A viral metagenomics pipeline, including quality controls, and taxonomic classification was applied as previously described [29]. Briefly, adapters and low-quality bases from 5′ and 3′ ends were trimmed using fastp v.0.20.0 [30], and low complexity reads and those shorter than 40 bases were removed. Exact duplicate reads were excluded using CD-HIT-DUP v.4.8.1 [31]. Ribosomal RNA and human-derived reads were removed by aligning against ribosomal sequences from SILVA database (DB) [32] and human genomes sequences from GenBank, respectively, using Bowtie2 v.2.3.4.3 [33]. The remaining reads were used for downstream analyses. For the taxonomic classification, valid reads were mapped against a viral reference nt DB (minimally non-redundant nucleotide DB) from NCBI (NCBI Resource Coordinators 2020), using SMALT v.0.7.6 (https://www.sanger.ac.uk/tool/smalt-0/) at 70% of identity. Mapped reads were assembled using IDBA v.1.1.3 [34], and contigs and unassembled singleton reads, were compared against all nt DB using BLASTn [35] to remove false positives. Non-mapping reads were assembled using IDBA, and contigs longer than 200 nt were compared to all proteins in NCBI’s nr DB (minimally non-redundant protein database) using BLASTx. The top 20 hits were considered from the resulting alignments. Then, MEGAN 6.21.2 [36] was used to taxonomically assign reads and contigs using its last common ancestor algorithm. Finally, taxa (viral species) with less than three assigned reads, seen in less than three samples each, were eliminated to reduce viruses that could be false positives.

### Differential virus abundance analysis

To compare groups of patients, read counts were normalized to reads per million (RPM) at 10 million reads to reduce differences due to uneven sequencing depths. Then, viral species that were differentially abundant in the various groups were identified with EdgeR v3.13 [37], using trimmed mean of M-values with singleton pairing (TMMwsp) normalization, which is the preferred method for data with a high proportion of zeros (high data sparsity), followed by dispersion estimation using the CR method with an offset of 0.01. Pairwise comparisons were carried out using a negative binomial GLM with quasi-likelihood tests. Only taxa with differences in associated FDR-adjusted p-values $\le$ 0.05 in at least one comparison were kept.

### Multivariate group analyses

RPM tables, described above, were also used for multivariate analyses with the R package vegan v2.5-7 [38]. The Bray–Curtis semi-metric was used to calculate weighted distances between each pair of samples. The resulting dissimilarity matrix (DM) was used to assess group variation with a PERMANOVA test (adonis) and a Multivariate homogeneity of groups dispersions test (betadisper). Three-way (ambulatory-hospitalized-deceased) group comparison and pairwise post-hoc adonis and betadisper tests for all pairwise group permutations were performed. The DM was also subjected to multi-dimensional scaling using distance-based redundancy analysis (dbRDA) with vegan’s capscale function. Since three groups were present, only two constrained linear combinations were created based on the type of patient.

### Assembly of viral genomes

For each viral species with high genomic coverage, the corresponding reference genome sequence was downloaded from GenBank to map all reads using Bowtie2 v2.3.4.3 [33]. Mapped reads were used to perform de novo assembly using SPADES v3.13.0 [39]. In case complete genomes were not obtained, consensus sequences were generated with iVar (v1.3.1) [40], using Phred score Q > 20 and a minimum read coverage depth of 5X to call a base of N for lower values. A threshold of at least 55% of the majority base rule was used. Finally, each contig or complete genome was verified using Blast v2.9.1 [35] against its corresponding reference.

## Results

### Sample collection and generation of SARS-CoV-2 genome sequences

We characterized the virome of SARS-CoV-2 positive samples from 120 patients, categorized into three different clinical outcome groups; ambulatory (36 samples), hospitalized (26 samples), and deceased (58 samples) (Table 1). Out of these, 41.7% of the participants had at least one comorbidity (Table 1 and Additional file 1: Table S1). As expected, a higher proportion of comorbidities were present in deceased patients (67.2%), with 40.4% having more than one comorbidity. Also, 38.5% of hospitalized patients presented comorbidities (11.5% having more than one), while only 2.8% of persons in the ambulatory group had comorbidity.

**Table 1:** Categories of SARS-2 positive samples used in this study

	All samples	Deceased	Severe	Ambulatory
Total	120^a^	58	26	36
No comorbidities	70	19	16	35
Comorbidities^b^	50 (41.7%)	39 (67.2%)	10 (38.5%)	1 (2.8%)
1 comorbidity	24 (20%)	16 (27.6%)	7 (27%)	1 (2.8%)
≥ 2 comorbidities	26 (21.7%)	23 (40.4%)	3 (11.5%)	0

^a^Number of samples in each category

^b^samples from patients with comorbidities were divided into two groups; with 1 comorbidity, and with 2 or more comorbidities

Conditions considered comorbidity: Obesity BMI index > 30, smoker, high blood pressure, diabetes, immunosuppression, HIV, COPD, kidney insufficiency, asthma, chronic disease

The age of patients varied across groups depending on the symptomatology (Additional file 2: Figure S1). Despite extant outliers, deceased patients comprised the most homogeneous set (Median = 36; IQR = 8) compared to hospitalized (Median = 41; IQR = 23.5) and ambulatory patients (Median = 36; IQR = 17). Regardless, no statistically significant difference was observed between any pairwise group permutations (Mann–Whitney; α = 0.05).

The presence of other respiratory pathogens was tested by RT-PCR assay in 19 samples for diagnostic purposes before metagenomic sequencing. No sample was found positive for any respiratory virus other than SARS-CoV-2 (Additional file 1: Table S1). Randomly amplified libraries for high-throughput sequencing were successfully prepared from all samples. In total, we obtained 3,486,527,880 paired-end reads, ranging from 3.6 × 10^6^ up to 77 × 10^6^ paired-end reads per sample (Additional file 1: Table S2). Out of 120 samples used, we obtained 70 complete SARS-CoV-2 genome sequences (> 98% genome coverage). In addition, 15 samples had a genome coverage of > 50%, nine samples had a coverage of 10 to 50%, and we could obtain only isolated SARS-CoV-2 reads from the remaining 26 samples (less than 10% coverage) (Additional file 1: Table S2). Whole genome sequences of SARS-CoV-2 generated in this work are described in more detail elsewhere and will not be discussed further in this work [41].

### Respiratory and other human viruses

Out of 120 samples analysed, 13 human respiratory viruses other than SARS-CoV-2 were found in 30 samples (25%). Five of these samples were from ambulatory patients, seven from hospitalized patients, and 18 samples from deceased patients (Additional file 1: Table S3). Nine samples (7.5%) harbored two additional respiratory viruses other than SARS-CoV-2; two were from ambulatory cases, one from a severe disease patient, and the six remaining samples were from deceased persons (Additional file 1: Table S3). The most common non-SARS-CoV-2 respiratory virus found in the samples was *Human mastadenovirus C*, which was found in 20 samples (16.7%) (Table 2, Additional file 1: Table S3), albeit always in low abundance (mostly < 100 reads per sample); *Human coronavirus HKU1* and *Rhinovirus B* were found in four samples; *Influenza B virus* was identified in two samples, and the remaining viruses were single occurrences (Table 2). None of the samples tested for other respiratory viruses by RT-PCR contained sequence reads similar to the viral species tested.

**Table 2:**
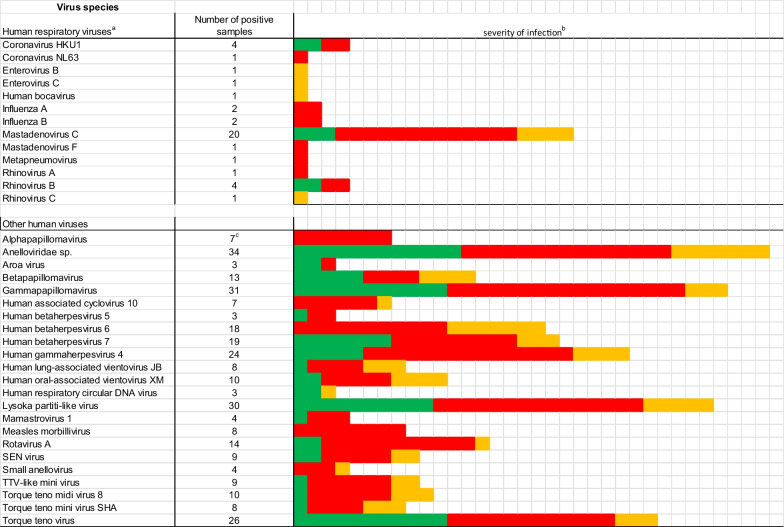
Presence of human viruses in SARS-2 positive samples

^a^Human viruses known to cause respiratory illness

^b^Severity of infection: red squares, deceased patients; orange, hospitalized patients; green, ambulatory cases

^c^Only viruses present in minimum of three samples are shown

**Fig. 1:**
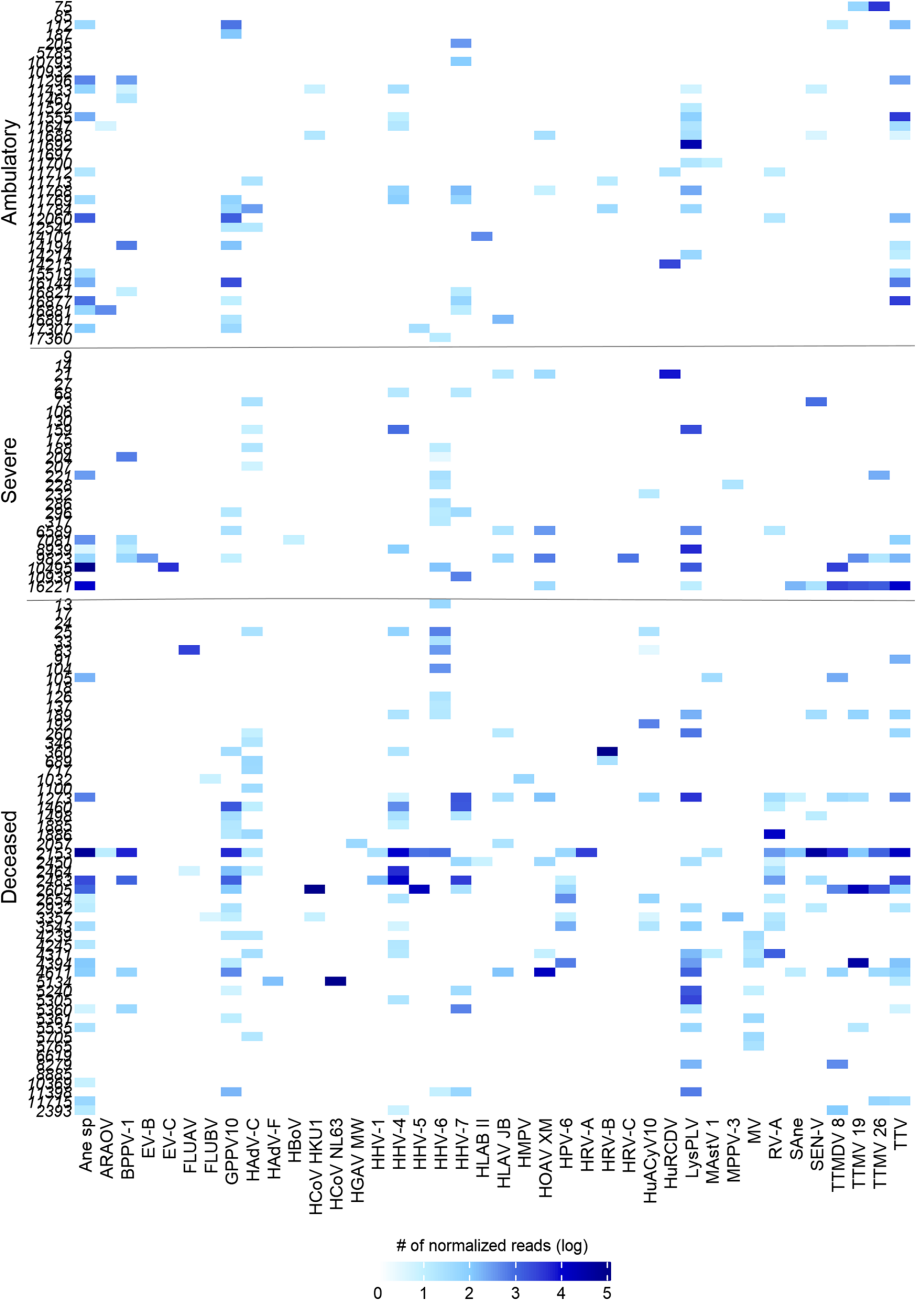
Abundance of human viral species in SARS-CoV-2 positive samples. Sequence reads were normalized according to the total number of reads after quality filtering. The abundance is shown in logarithmic scale (log10). Samples from ambulatory, severe and deceased patients are separated vertically

Interestingly, reads corresponding to at least one human non-respiratory virus were found in 97 samples (80.8%) (Additional file 1: Table S4). They were present in 31 out of 36 ambulatory cases (86.1%), 20 of 26 hospitalized patients (76.9%), and 46 out of 56 deceased patients (82.1%). In total, we have identified 27 additional human non-respiratory viruses belonging to 10 viral families, with four viruses being unclassified at the family level. Among the most prevalent were members of the *Anneloviridae* family, identified in 37% of the samples, with seven viral species, followed by viruses in the family *Herpesviridae*, found in 41.7% of the samples, with five species, and viruses in the *Papillomaviridae* family, in 34.2% of the samples, with four species (Fig. 1; Additional file 1: Table S4). Other reads belonging to viruses in families *Picobirnavirida*e (25% of prevalence), *Reoviridae* (10.8%), *Circoviridae* (8.3%), *Paramyxoviridae* (6.7%), *Astrovirida*e (3.3%), *Flaviviridae* (2.5%) and unclassified viral sequences (11.7%), were also identified in some samples. One sample (number 383 from a deceased patient without comorbidities) did not present any other viral reads than SARS-CoV-2 and was excluded from further analyses.

Apart from viruses known to infect humans, we identified many sequences that showed similarity to viruses that infect other organisms, including animal species, plants, invertebrates, yeast, and amoebas (Additional file 1: Tables S5 and S6). These results are not discussed further in this work and will be addressed in future studies.

### Some virus species are associated with different severity of disease groups

We have observed that some viral species were present predominantly in some of the severity groups analysed in this study. Table 3 shows the viral species that were differentially distributed among paired study groups (pairwise comparisons of samples from ambulatory, hospitalized, and deceased patients). Eight viral species were significantly more prevalent in deceased patients compared to ambulatory ones: *Torque teno midi virus 8* (TTMDV 8), *TTV-like mini virus 19* (TTMV 19), *Torque teno mini virus SHA* (TTMV 26), *Human betaherpesvirus 6* (HHV 6), *Human associated acyclovir 10* (HuACyV 10), *Rotavirus A* (RV-A), *Measles morbillivirus* (MV) and *Alphapapilomavirus 10* (HPV-6). Likewise, five of these species were also more prevalent in hospitalized than in ambulatory patients (TTMDV 8, TTMV 19, TTMV 26, HHV 6, and HuACyV 10). Interestingly, there was also different distribution of some of these human viral species when samples from deceased patients were compared to samples from hospitalized patients, with three being more prevalent (RV-A, MV, and HPV 6). All other group comparisons showed no statistically significant differences for other viruses.

**Table 3:** Virus species differentially abundant in patients with diverse severity of infection

Viral species	All samples	Number of positive samples			Comparing dec/amb		Comparing hosp/amb		Comparing dec/hosp	
		Deceased	Hospitalized	Ambulatory						
	120	57	26	37	lfch^a^	FDR^b^	lfch	FDR	lfch	FDR
TTMDV 8	10	6	3	1	**4.201**^**c**^	**0.025**	**4.279**	**0.021**	0.078	0.97
TTMV 19	9	6	2	1	**16.118**	**3.04E-07**	**9.947**	**0.0004**	− 6.171	0.093
TTMV 26	8	4	3	1	**4.815**	**0.003**	**4.298**	**0.021**	− 0.517	0.944
HHV-6	19	12	7	0	**9.986**	**1.98E-06**	**11.361**	**1.24E-06**	1.374	0.595
HuACyV10	8	7	1	0	**12.793**	**1.07E-06**	**13.305**	**1.81E-06**	0.511	0.944
RVA	14	11	1	2	**4.516**	**0.013**	− 1.925	0.395	− **6.442**	**0.016**
MV	8	8	0	0	**14.037**	**4.90E-07**	0	1	− **14.037**	**0.0005**
HPV-6	7	7	0	0	**14.851**	**4.90E-07**	0	1	− **14.851**	**0.00003**

^a^log fold change

^b^FDR represents the p-value adjusted by FDR using Benjamini–Hochberg correction

^c^Significant results are in bold

**Fig. 2:**
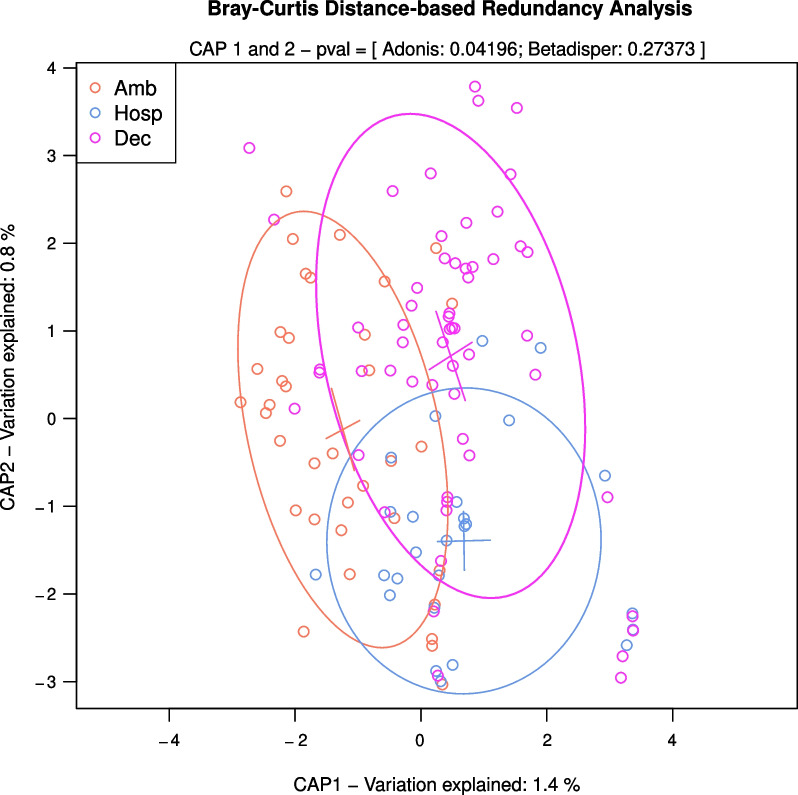
Distance-based analysis of human virus communities among SARS-CoV-2 positive samples according to severity groups. The Analysis of Principal Coordinates (cap linear combination) is based on Bray Curtis-derived distance metric calculated on the abundance of human viruses in samples classified by the type of patient. Axes show the two Constrained Analysis of Principal Coordinates (CAP linear combinations) which are coerced by the type of patient. These account for 2.2% of total variation. Each sample is represented with a circle and distances show how similar they are from one another. Ellipses show within-group variation with a 0.75 confidence limit based on the standard deviation. Red circles represent ambulatory samples, blue represent hospitalized patients and fuchsia represents samples from deceased patients

To further evaluate the differences in the composition of viral communities in samples from different groups of patients, Bray–Curtis dissimilarities distances were calculated using the relative abundance of human viruses, and statistical comparisons were carried out by attribute and in pairwise post hoc analyses (Fig. 2 shows the resulting dbRDA from the human virus matrix). These results indicated that differences between ambulatory patients and the other groups were significant (PERMANOVA for ambulatory-deceased p-value = 0.03, ambulatory-hospitalized p-value = 0.04), but the difference between the hospitalized and deceased patients was not statistically significant (p-value = 0.49). We also corroborated that the variances between groups were comparable (betadisper p-value = 0.2827). Furthermore, other features, such as comorbidities, sex, and sampling location, were compared between groups, but no significant differences were observed (results not shown).

### Generating complete genomes of other viruses

Our strategy allowed us to obtain enough viral reads in some of the samples to assemble complete or partial genomes of other viruses, in addition to SARS-CoV-2. We could assemble at least 25% of the genome for 70 viruses other than SARS-CoV-2 belonging to 14 different families, with three viruses being unclassified (listed in Additional file 1: Table S7). The most common family with complete genomes was *Anelloviridae*, found in 16 samples, and out of 28 anelloviruses, seven had a genome coverage > 90%. Interestingly, we assembled the complete genome of two other coronavirus species (*Human coronavirus HKU1* and *Human coronavirus NL63* in one sample each) and one other respiratory virus (Human rhinovirus B3 in one sample). The remaining viruses with > 25% genome coverage belong to different groups; human viruses (diverse papillomaviruses, human-associated cyclovirus, gut, and oral-associated vientovirus, circular DNA viruses, and enterovirus B), and diverse plant viruses.

## Discussion

Data regarding SARS-CoV-2 coinfections with other viruses and the role these viruses could have in the severity of the disease are still limited. Most of the previous studies have used RT-qPCR assays focused on detecting a few specific respiratory viruses [11, 12, 14–18, 27], and only some studies have used metagenomic approaches [21–24, 42]. In this study, we used sequence-independent metagenomic sequencing to determine the whole virome in three different severity groups of patients (deceased, hospitalized, and ambulatory) infected with SARS-CoV-2 and to explore whether there could be a correlation between the presence of viruses other than SARS-CoV-2, and severity of the disease. Despite processing samples to decrease the amount of contaminant genetic material, all samples still contained human reads. This is standard in viromic studies, suggesting that filtration and nuclease treatment were not absolute.

We found that 25% of the 120 samples analysed were positive for another respiratory virus than SARS-CoV-2, with 7.5% having two additional respiratory viruses. This high level of co-occurrence has been previously observed in other studies: 20.7% in North California [16], 28.4% in patients from Jiangsu Province, China [19], and 45% in patients in Iran [12]. Previously, rhinoviruses/enteroviruses have been found as the predominant coinfecting agents [16, 20, 27], with influenza being frequent in one study [12]. In this study, we identified Human mastadenovirus C as the most prevalent, being present in 20 samples (16.7%), although with low abundance (< 100 reads per sample). This high level of co-occurrence with Human mastadenovirus C was not expected, as it is not recognized as a frequent pathogen in respiratory diseases, generally being found in less than 10% of samples of children under five years of age, without a clear seasonality, and instead all year around [43, 44]. Human mastadenovirus C had been reported in lower prevalence (4.8%) in SARS-CoV-2 positive samples in Brazil [45]. Regarding other respiratory viruses, similar to most other published works, [11, 16, 18, 27, 45], we observed Influenza A or B viruses in only a few samples. These low co-occurrence values in our samples could be explained by the time of sample collection, as the influenza season in Mexico generally ends in March–April, and due to the anti-SARS-CoV-2 preventive measures implemented (wearing masks, social distancing) and the anti-influenza vaccination campaign in Mexico. We found *Human coronavirus HKU1* and *Rhinovirus B* in four samples each (3.3%), and other respiratory viruses were found in only one or two samples.

The presence of other human non-respiratory viruses in SARS-CoV-2 positive samples has been even less studied during SARS-CoV-2 infections. A study by Kim and collaborators [22] reported a high prevalence of other non-respiratory viruses in southern hemisphere samples (74%, 68 out of 92 samples), while another metagenomic next-generation sequencing (mNGS) work did not present a detailed virome composition [21, 23, 24]. Our study identified other (non-respiratory) human viruses in 97 out of 120 samples (80.8%). We found sequences with homology to 27 different viruses (Additional file 1, Table S4). The viruses most frequently detected in our study differ from those identified by Kim et al. [22]. They reported mammarenaviruses, rodeoloviruses, and alphapolyomaviruses as the most frequent, followed by papillomaviruses and lymphocryptoviruses. Recent virome analysis of samples collected in Italy has identified six viral families in SARS_CoV-2 samples (*Retroviridae, Herpesviridae, Poxviridae, Pneumoviridae, Anelloviridae, and Pandoraviridae*), with the first three families being most prevalent [46]. In our study, the most common families found were *Annelloviridae*, with seven members, *Herpesviridae* with five species, and *Papilomaviridae,* with four viral species. Differences observed may be due to geographic locations and differences in the populations studied. Regarding the evolution of the patients’ disease, significant differences were found when the abundance of human viruses was compared between types of patients globally, as well as between ambulatory and hospitalized patients, and between ambulatory and deceased patients; no statistical significance was found in differences between hospitalized and deceased patients. When observing the virus species that could contribute to these differences, some were preferentially abundant in samples from deceased or/and hospitalized patients (Table 3); however, given the type of viruses identified and fact that they were only present in a small proportion of all patients, their association with the severity of the infection cannot be established, but rather, those viruses that were present, showed higher sample frequency and overall abundance in hospitalized or deceased patients.

For example, in this study, some Anelloviruses were identified in high frequency, principally in hospitalized and deceased patients. These are ubiquitous viruses that persistently and commonly infect humans [47], which could explain their frequent presence in samples. Alternatively, their presence could reflect an increased replication due to alterations of the immune responses during SARS-CoV-2 infection, as described for other infections [48, 49], and increased coinfection in severe cases of disease would be result of SARS-CoV-2 infection. In the case of measles virus, which in this work was identified abundantly and frequently in deceased patients, it has been described that its infection could cause immune suppression [50], which could lead to more severe disease. However, the impact of other coinfecting viruses on the COVID-19 severity remains to be explored. Recently, Paparoupa and collaborators [13] described an increase in coinfection of SARS-CoV-2 positive patients on invasive ventilation with *Herpes simplex virus* and *Cytomegalovirus*, possibly as a result of long treatment, suggesting that time spent in the hospital could have an effect on the viral presence, which is consistent with HHV6 and HuACyV 10 viruses being identified in higher frequency and abundance in deceased patients which had been hospitalized.

When analysing the presence of other respiratory viruses in deceased patients from North Khorasan (Iran), a high level of coinfection between SARS-CoV-2 and Influenza A virus (22.3% of the samples) was observed [12]. Furthermore, Respiratory syncytial virus (RSV) and bocavirus were each present in 9.7% of the samples. Although that work focused only on samples from deceased patients, without comparing the results with other populations, for example, ambulatory patients, it is tempting to speculate that coinfection with *Influenza A virus* and RSV (two respiratory pathogens that are the cause of severe disease) could play a role in the high mortality reported. Coinfection with Influenza and adenoviruses has also been reported to be significantly associated with increased mechanical ventilation (influenza viruses) or death (both viruses) [51]. Moreover, two recent studies in mouse and ferret models demonstrated that SARS-CoV-2 and *Influenza A virus* coinfection causes more severe pathology [52, 53]. In our study, these coinfections were rare for influenza viruses and null for RSV. Concerning adenoviruses homologs to *Mastadenovirus C* and *F* were frequent but could not be associated with pathogenicity. It is of interest that, in our case whole genome of three different respiratory viruses (human rhinovirus B and human coronaviruses HKU1 and NL63) were obtained from samples corresponding to deceased patients without comorbidities. Viral metagenomics approaches are considered semi-quantitative, and the ability to assemble whole viral genomes suggests a high load of a given virus in the sample, most probably due to an active infection rather than the presence of a virus that is just passing through. However, it is not known whether these coinfections had an impact on disease severity.

Our study has several limitations. Firstly, our samples were from patients with and without different comorbidities (Additional file 1: Table S1), making it difficult to draw conclusions concerning the role of other viruses on the disease outcome. Furthermore, samples were derived from nasopharyngeal or oropharyngeal swabs or from tracheal aspirates, and the sample type may affect virus detection [54, 55]. Finally, all the samples analysed were from SARS-CoV-2 infected subjects, and a group of SARS-CoV-2 negative patients was not included. These inconsistencies among the samples (presence of comorbidities and different types of samples), together with the expected diversity of the inter-individual respiratory virome [56], make it difficult to observe differences among viral communities in samples from different study groups (deceased, severe-hospitalized, and ambulatory cases). Despite these limitations, we were able to observe differences in the abundance of human viral species when samples from ambulatory, hospitalized, and deceased individuals were compared, being higher in severe and deceased persons as compared to ambulatory patients.

## Supplementary Information

**Additional file 1: Table S1.** Patients included in the study. Information of samples included in study, sample ID, age, and sex of patients, place of collection, date of sample and start of the symptoms, clinical status, comorbidities, sample type, and diagnostic test for other respiratory viruses if performed. **Table S2.** General sequencing data and SARS-CoV-2 sequenced genome characteristics. Number of reads (original total reads, filtered reads, SARS-CoV-2-specific reads, reads used for metagenomic analysis) and results of SARS-CoV-2 genome sequencing. **Table S3.** Respiratory viruses found in SARS-CoV-2 positive samples. List of samples with homologs to other (non-SARS-CoV-2) human respiratory viruses. **Table S4.** Other human (non-respiratory) viruses, found in SARS-CoV-2 positive samples. List of samples with homologs to other non-respiratory human viruses. **Table S5.** Viruses of other vertebrates found in SARS-CoV-2 infected patients. Contains list of samples with homologs to viruses of other vertebrate species found in SARS-CoV-2 infected patients. **Table S6.** Non-vertebrate viruses found in SARS-CoV-2 infected persons. Contains list of samples with homologs to non-vertebrate viruses. **Table S7.** Non-SARS-CoV-2 viruses with > 25% genome coverage. Contains list of viruses, where more than 25% genome was sequenced, from those found in SARS-CoV-2 positive samples.

**Additional file 2: Figure S1.** Age distribution in different outcome groups. Boxplot shows age quartiles for each group of patients (deceased, hospitalized and ambulatory). Boxes are delimited by Q_1_ and Q_3_ and whiskers show IQR*1.5 from either Q_1_ or Q_3_. Medians (Q_2_) are shown as black horizontal bars inside boxes.

## Data Availability

Sequences are readily available at NCBI’s SRA under project PRJNA819439 with accession numbers: SRR19400449-SRR19400568. Available at: https://www.ncbi.nlm.nih.gov/bioproject/PRJNA819439/.

## Abbreviations

SARS-CoV-2: Severe acute respiratory syndrome coronavirus 2
COVID-19: Coronavirus disease 2019
RT-PCR: Reverse transcription polymerase chain reaction
InDRE: Instituto de Diagnóstico y Referencia Epidemiológicos
INER: Instituto Nacional de Enfermedades Respiratorias
RNLSP: Red Nacional de Laboratorios Estatales de Salud Pública
IMSS: Instituto Mexicano del Seguro Social
WHO: World Health Organization
RNA: Ribonucleic acid
cDNA: Complimentary deoxyribonucleic acid
DB: Data bank
NCBI: National Center for Biotechnology Information
RPM: Reads per million
DM: Dissimilarity matrix
dbRDA: Distance-based redundancy analysis
PCoA/NMDS: Principal coordinates analysis and multidimensional scaling
TTMDV 8: Torque teno midi virus 8
TTMV 19: TTV-like mini virus 19
TTMV 26: Torque teno mini virus SHA
HHV 6: Human betaherpesvirus 6
HuACyV 10: Human associated cyclovirus 10
RV-A: Rotavirus A
MV: Measles morbillivirus
HPV-6: Alphapapilomavirus 10
RT-qPCR: Reverse transcription quantitative polymerase chain reaction
mNGS: Metagenomic next-generation sequencing
RSV: Respiratory syncytial virus
