# Supplementary figures and images for: Metagenomic analysis reveals differences in the co-occurrence and abundance of viral species in SARS-CoV-2 patients with different severity of disease
Source: BMC Infect Dis. 2022 Oct 19;22:792. doi: 10.1186/s12879-022-07783-8 (PMC9580447; doi:10.1186/s12879-022-07783-8)

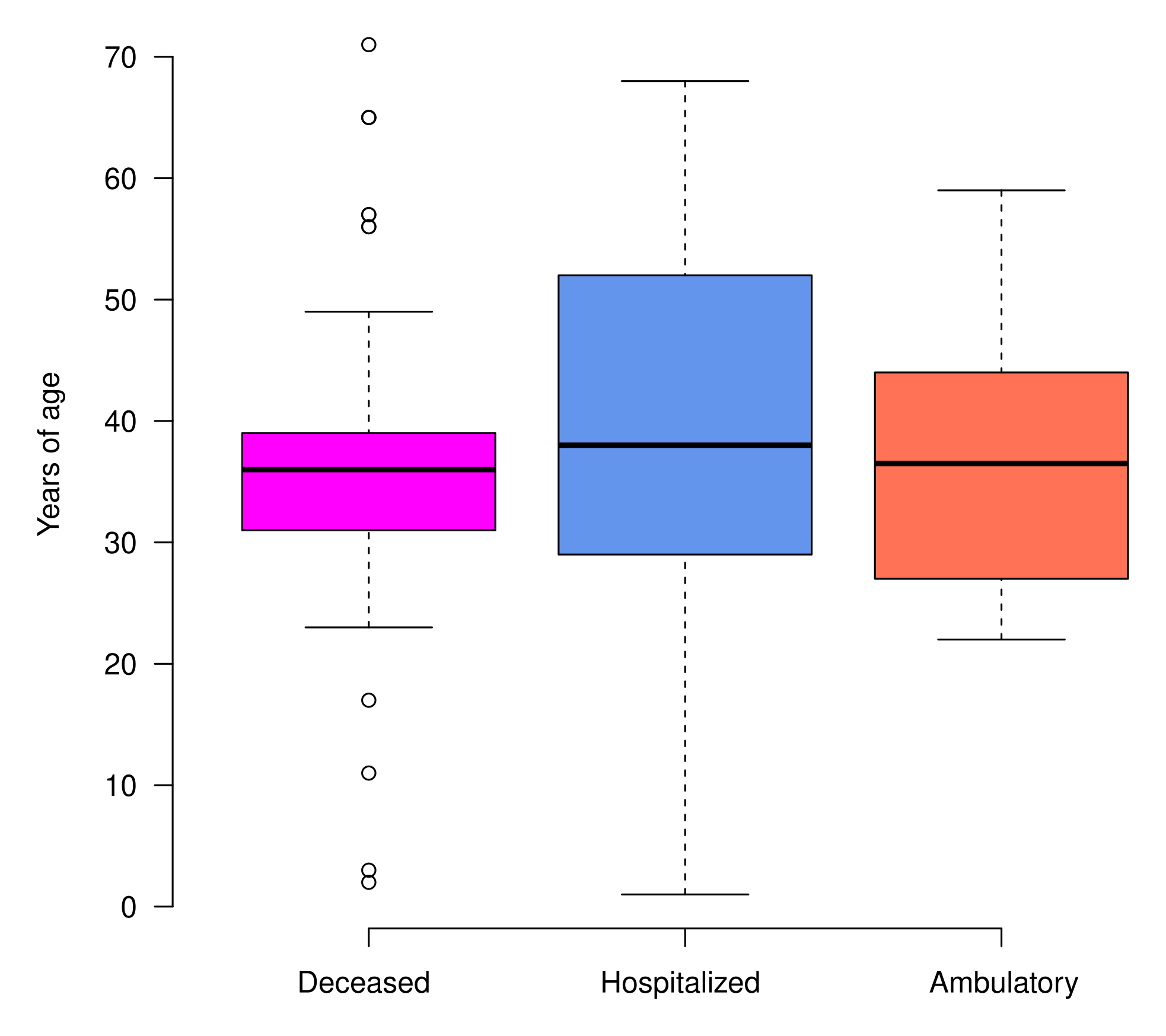

Supplement: Supplementary file 2 — Additional file 2: Figure S1. Age distribution in different outcome groups. Boxplot shows age quartiles for each group of patients (deceased, hospitalized and ambulatory). Boxes are delimited by Q1 and Q3 and whiskers show IQR*1.5 from either Q1 or Q3. Medians (Q2) are shown as black horizontal bars inside boxes. [file 12879_2022_7783_MOESM2_ESM.tif]
